# Impact of Psychiatric History on Healthcare Delivery

**DOI:** 10.1192/j.eurpsy.2025.1571

**Published:** 2025-08-26

**Authors:** A. Castelao Escobar, E. Gil Benito, P. Sanz Sánchez, R. González Nuñez, C. Alguacil Núñez, R. Albillos Pérez

**Affiliations:** 1 Psiquiatría, Hospital Universitario Puerta de Hierro Majadahonda; 2 Psiquiatría, Hospital Universitario El Escorial, Madrid, Spain

## Abstract

**Introduction:**

A history of psychiatric disorders significantly impacts patient evaluation and treatment in healthcare. Nearly 80% of excess mortality in individuals with mental illnesses is due to physical health issues. While suicide risk is widely recognized, most mortality from mental illnesses stems from physical conditions like cardiovascular diseases, respiratory issues, and cancer, leading to a life expectancy reduction of 15-20 years compared to the general population.

**Objectives:**

To exemplify through a case the possible impact of psychiatric history on healthcare provision.

**Methods:**

We present the case of a 55-year-old woman with a history of severe functional neurological disorder resulting in mobility limitations, dyskinetic movements, and functional pain characterized as allodynia. She has been followed by neurology for the past year. She began mental health follow-up one year earlier due to reactive anxiety stemming from work-related issues. Her substance use history includes being a tobacco smoker for over 20 years (FTND score>50) and a former consumer of three liters of beer daily for two years, with periods of abstinence. The patient presented to the emergency department due to weight loss and low mood, worsening over the past few weeks, in the context of intense muscle pain and loss of strength. Blood tests and a brain CT scan were performed without abnormalities. She was initially admitted to Neurology for severe functional neurological disorder for symptom control, but was later transferred to Psychiatry due to suicidal threats. During her psychiatric admission, she experienced improvement in pain and subsequently in her mood. After discharge from psychiatry, the patient returned several times to the emergency department due to worsening neurological symptoms, being discharged with a diagnosis of functional disorder.

**Results:**

Two months after her discharge from psychiatry, the patient returned to the emergency department due to rib pain. After emergency assessment, she was referred to Neurology for worsening neurological symptoms. With suspicion of paraneoplastic polyneuropathy, an MRI of the brain and spine, EMG, lumbar puncture, and CT scans of the neck, chest, abdomen, and pelvis were performed during her neurology admission. She was diagnosed with small cell lung carcinoma stage IVA and asymmetric paraneoplastic sensory neuropathy. Following the diagnosis, she was referred to Oncology, receiving one cycle of chemotherapy. However, given the unfavorable progression, it was decided, in agreement with the patient, not to continue active oncological treatment, and she was transferred to a Palliative Care Center.

**Conclusions:**

This case emphasizes the need to recognize how psychiatric history affects medical care. Stigma and the patient’s challenges in communicating symptoms can hinder the diagnosis and treatment of organic conditions, contributing to the excess mortality associated with mental health issues.

**Disclosure of Interest:**

None Declared

